# Validation of a Kazakhstani Version of the Mental Health Continuum—Short Form

**DOI:** 10.3389/fpsyg.2021.754236

**Published:** 2021-10-13

**Authors:** Daniel Hernández-Torrano, Laura Ibrayeva, Ainur Muratkyzy, Natalya Lim, Yerden Nurtayev, Ainur Almukhambetova, Alessandra Clementi, Jason Sparks

**Affiliations:** ^1^Graduate School of Education, Nazarbayev University, Nur-Sultan, Kazakhstan; ^2^Department of Medicine, Nazarbayev University School of Medicine, Nur-Sultan, Kazakhstan; ^3^Health and Wellness Center, Nazarbayev University, Nur-Sultan, Kazakhstan

**Keywords:** mental health, well-being, MHC-SF, health psychology, validation, psychometric adequacy, Kazakhstan, Central Asia

## Abstract

Positive mental health and well-being are significant dimensions of health, employment, and educational outcomes. Research on positive mental health and well-being requires measurement instruments in native languages for use in local contexts and target populations. This study examines the psychometric properties of the Kazakhstani version of the Mental Health Continuum—Short Form (MHC-SF), a brief self-report instrument measuring emotional, social, and psychological well-being. The sample included 664 University students (425 females) purposefully selected in three higher education institutions in South, East, and Central Kazakhstan. Their average age was 20.25 and ranged from 18 to 43. Participants completed a Kazakhstani version of the MHC-SF online. Statistical analyses to evaluate the structural validity, reliability, and measurement invariance of the Kazakhstani version of the MHC-SF were performed. The results confirmed the superiority of the bifactor model (i.e., three separated factors of well-being plus a general factor of well-being) over the alternatives. However, most of the reliable variance was attributable to the general well-being factor. Subscale scores were unreliable, explaining very low variance beyond that explained by the general factor. The findings demonstrated the measurement invariance of the MHC-SF across gender and age. Overall, these findings support the use of the Kazakhstani version of the MHC-SF to examine a general factor of well-being and the measurement invariance of the instrument across gender and age groups. However, the results advise against the interpretation of the subscale scores as unequivocal indicators of emotional, social, and psychological well-being.

## Introduction

The WHO defines mental health as “a state of well-being in which every individual realizes his or her own potential, can cope with the normal stresses of life, can work productively and fruitfully, and is able to make a contribution to her or his community” (World Health Organization, 2004, p. 12). This definition breaks with a tradition based on psychopathological-oriented models of mental health with a focus on disorders and illness toward a conceptualization that pays more attention to the presence of positive features and what is right about people (Kobau et al., 2011). It builds upon two long-standing traditions in positive psychology: hedonic and eudaimonic well-being. The hedonic approach is connected to happiness and defines well-being as the presence of positive feelings and pleasure and the absence of negative feelings or pain. The eudaimonic approach focuses on self-realization and meaning and defines well-being as the degree to which a person is functioning appropriately (Ryan and Deci, 2001; Keyes, 2006, 2007; Jovanović, 2015; Joshanloo and Lamers, 2016).

International interest in positive mental health and well-being has grown exponentially in recent decades due to the significant effects they have on health, employment, and educational outcomes (see Keyes, 2013, for an international review of correlates to mental well-being in these domains). Research on positive mental health and well-being requires measurement instruments that provide reliable and valid scores in different contexts, cultures, and languages. The Mental Health Continuum—Short Form (MHC-SF) (Keyes et al., 2008) is one of the most widely used self-report instruments to measure positive mental health and well-being around the world in clinical (e.g., Silverman et al., 2018; van Erp Taalman Kip and Hutschemaekers, 2018; Donnelly et al., 2019), work (e.g., Jaotombo, 2019), and educational settings (e.g., de Carvalho et al., 2016; Luijten et al., 2019).

The MHC-SF is a 14-item measure of positive mental health that encompasses both hedonic and eudaimonic well-being (Keyes et al., 2008). It was designed to measure three dimensions of positive mental health: (1) emotional well-being, which refers to the presence of positive feelings and life satisfaction; (2) social well-being, which accounts for adequate social functioning and connection to society; and (3) psychological well-being, which reflects personal functioning and thriving in life. In the MHC-SF, emotional well-being, social well-being and psychological well-being are measured using three, five, and six items, respectively. Emotional well-being reflects the hedonic approach to well-being, whereas social and psychological well-being are used as indicators of eudaimonic well-being (see Lamers et al., 2011; Petrillo et al., 2015).

Along with these three dimensions of well-being, the MHC-SF can also help categorize three states of mental health: flourishing, moderate mental health, and languishing (Keyes, 2002). Flourishing represents high levels of well-being in both hedonic and eudaimonic well-being. Languishing in a mental health state characterized by lower scores in hedonic and eudaimonic well-being. Individuals who do not meet the criteria for either flourishing and languishing are considered to demonstrate moderate levels of mental health (Keyes, 2005). The results of the first study using the MHC-SF demonstrated that 12.2% of the participants were languishing, 67.8% were moderately mentally healthy, and 20% were flourishing (Keyes et al., 2008). Other studies with adult populations have found similar distributions of the categorical diagnosis of states of mental health, with 10–20% of participants languishing, 50–70% moderately mentally healthy, and 20–30% flourishing (e.g., Lamers et al., 2011; Petrillo et al., 2015), although there is wide variability across contexts. Younger samples such as adolescents and college students tend to demonstrate comparatively higher distributions of flourishing and moderately mentally healthy (e.g., Luijten et al., 2019; Hides et al., 2020).

The psychometric characteristics of the MHC-SF have been widely explored across various contexts, cultures, and languages, predominantly in Europe (e.g., Lamers et al., 2011; Karaś et al., 2014; Jovanović, 2015; Petrillo et al., 2015; Joshanloo and Lamers, 2016; Echeverría et al., 2017; Joshanloo and Jovanović, 2017; Donnelly et al., 2019; Luijten et al., 2019; Santini et al., 2020; Monteiro et al., 2021). Evidence about the appropriate reliability and validity of translated versions of the scale also exists in other continents, including Asia (Lim, 2014; Guo et al., 2015; Rafiey et al., 2017; Rogoza et al., 2018; Joshanloo, 2020), North America (Joshanloo, 2016a, 2018, 2019; Lamborn et al., 2018), South America (Contreras et al., 2017; Perugini et al., 2017), Africa (Keyes et al., 2008; De Bruin and Du Plessis, 2015), and Oceania (Hides et al., 2016; Joshanloo et al., 2017). Such studies are paramount because measuring instruments that have been developed and normed in one context (e.g., Western; more individualistically socially oriented) must be translated and validated before they can be administered in other contexts (e.g., Asian/African; more collectivistically socially oriented). This is especially true in the field of mental health and well-being because the issues of the “mind” are often interpreted very differently across contexts and countries (Fernando, 2019). Indeed, cultural values, traditions, and languages influence conceptions of mental health and well-being (Eshun and Gurung, 2009; Vaillant, 2012). No previous study has examined the properties of this instrument in Central Asia. This study aimed to fill this gap by examining the reliability and structural validity of the Kazakhstani version of the MHC-SF.

The factor structure of the MHC-SF has been extensively analyzed using exploratory factor analysis (EFA) (Lamers et al., 2011; Rafiey et al., 2017), confirmatory factor analysis (CFA; Karaś et al., 2014; Guo et al., 2015; Petrillo et al., 2015), and exploratory structural equation modeling (ESEM) (Joshanloo and Lamers, 2016; Joshanloo and Jovanović, 2017). Joshanloo (2018) has also recently examined the internal structure of the MHC-SF using a multidimensional scaling approach. Overall, the results of these studies support the original structure of the questionnaire, with three-factor models (i.e., emotional, social, and psychological well-being) demonstrating a better fit than one-factor (i.e., mental health), two-factor (i.e., hedonic and eudaimonic well-being), and second-order factor models (i.e., three correlated first-order factors representing emotional, social, and psychological well-being loading into a second-order general well-being factor).

However, there is a growing concern regarding the goodness of fit of the three-factor model. An alternative bifactor model has been recently proposed as a superior explanation of the internal structure of the MHC-SF (e.g., De Bruin and Du Plessis, 2015; Jovanović, 2015; Echeverría et al., 2017; Schutte and Wissing, 2017; Rogoza et al., 2018; Silverman et al., 2018; Longo et al., 2020). The bifactor model accounts for a general well-being factor and three separate well-being factors capturing specific variance for emotional, social, and psychological well-being. In general, these studies argue that the bifactor model fits the data better than alternative models. In the bifactor model, the general well-being factor accounts for a greater amount of variance than the three specific well-being factors, and after controlling for the variance of the general factor, the three specific factors explain a very little amount of variance. Moreover, a strong general factor of well-being seems to emerge in these studies, no matter whether a CFA bifactor or an ESEM bifactor approach are used (Longo et al., 2020).

Studies exploring the three-factor solution of the MHC-SF consistently report satisfactory internal consistency (Keyes et al., 2008; Lamers et al., 2011; Perugini et al., 2017; Luijten et al., 2019) and test-retest reliability (e.g., Petrillo et al., 2015). Cronbach's coefficient α for the general and specific subscale scores in the bifactor model seem to be also appropriate across studies (e.g., De Bruin and Du Plessis, 2015; Echeverría et al., 2017). However, when alternative reliability coefficients such as McDonald's ω are used to estimate the reliability of the scale scores in the bifactor model, the findings are less conclusive. In some cases, coefficients ω suggest good reliability of the scores for the general and separate well-being subscales (e.g., Lamborn et al., 2018; Rogoza et al., 2018). In other cases, findings suggest that the general factor tends to account for a greater amount of variance of the MHC-SF and that three subscales demonstrate low reliability and explain very little variance beyond that explained by the general factor (Jovanović, 2015; Echeverría et al., 2017; Schutte and Wissing, 2017; Silverman et al., 2018; Longo et al., 2020; Santini et al., 2020).

The measurement invariance of the MHC-SF has also been explored in the literature. In general, there is considerable evidence supporting the invariance of the structure of the MHC-SF across different groups, contexts, and conditions. Measurement invariance across gender and age has been observed in most studies for both the three-factor (e.g., Karaś et al., 2014; Guo et al., 2015; Joshanloo and Jovanović, 2017; Joshanloo et al., 2017; Perugini et al., 2017) and bifactor solutions (e.g., Echeverría et al., 2017; Lamborn et al., 2018) of the MHC-SF. The longitudinal measurement invariance of the bifactor model has also been examined, with no apparent differential item functioning over time (Lamers et al., 2011). Moreover, there is growing evidence for the full or partial cross-cultural measurement invariance of the instrument. For example, Joshanloo (2016a) demonstrated that the items of the MHC-SF function relatively similarly across samples in Iran and the USA when considering the three-factor model. Schutte and Wissing (2017) also reported evidence for the partial invariance of the bifactor model across three cultural groups in South Africa. Similarly, Zemojtel-Piotrowska et al. (2018) investigated the cross-cultural measurement invariance of the MHC-SF across 38 countries, confirming “the cross-cultural replicability of a bifactor structure” (p. 1035).

## Materials and Methods

### Participants

The sample included 664 University students purposefully selected in three higher education institutions located in South, East, and Central Kazakhstan. Among them, 425 were females (64.0%), 236 were males (35.5%), and the 3 did not report their gender (0.5%). Their age ranged from 18 to 43 (*M* = 20.25, *SD* = 3.61). A total of 383 were under 20 years old (57.7%), and 281 were 20 years-old or older (42.3%). Most of the participants were single and had no children. A majority of them were studying an undergraduate program (79.7%). The rest were enrolled in a master's program (11.9%), a PhD program (3.2%), or other programs (5.2%). From the total sample, 11% were studying a major in Natural Sciences, 49% in Technical Sciences, 30% in Social Sciences, and 10% in Humanities.

### Instrument and Procedures

The Mental Health Continuum-Short Form (MHC-SF) (Keyes et al., 2008) is a brief questionnaire that measures positive mental health. It comprises 14 items that represent several feelings of well-being: three items reflect emotional well-being (items 1–3) (e.g., In the past month, how often did you feel happy), five items reflect social well-being (items 4–8) (e.g., In the past month, how often did you feel that you had something important to contribute to society), and six items reflect psychological well-being (items 9–14) (e.g., In the past month, how often did you feel that you liked most parts of your personality). Participants rate the frequency of each feeling in the past month on a 6-point Likert scale (0 = never, 1 = once or twice, 2 = about once a week, 3 = about 2 or 3 times a week, 4= almost every day, 5 = every day). The MHC-SF offers two levels of assessment. First, it allows for the evaluation of the three well-being dimensions (i.e., emotional, social, and psychological). Second, a categorical diagnosis of mental health status with three categories: flourishing, moderate, and languishing.

The MHC-SF was translated into the two official languages of Kazakhstan (i.e., Russian and Kazakh) using a back-translation approach (Brislin, 1970). Two members of the research team who were native speakers translated the MHC-SF into Russian and Kazakh languages. Next, independent translators who were unfamiliar with the original version of the instrument translated these versions back to English language. The research team then examined the original and back translated versions to ensure comparability. In addition to that, the Russian and Kazakh translations of the MHC-SF were further assessed by the research team to ensure understandability, psychological equivalence, and the accuracy of the translations (Douglas and Craig, 2007).

The Kazakhstani version of the MHC-SF was distributed online via email by the gatekeepers of the respective universities. Participants provided informed consent before proceeding to complete the questionnaire. Anonymity and confidentiality were ensured and no information that could identify the identities of the participants was collected. The study was approved by the Ethics Committee of the authors' institution (reference number 195/19112019).

### Statistical Analysis

Descriptive statistics were calculated to examine the distribution of the scores. Relevant subscales items were summed to yield a score for emotional well-being (items 1–3; range 0–15), social well-being (items 4–8; range 0–25), and psychological well-being (items 9–14; range 0–30). A total well-being score was obtained by summing up the 14 items of the scale (range 0–70). The categorical diagnosis using the MHC-SF by Keyes (2006) was applied to the data to obtain estimates of the population prevalence of the mental health categories. A diagnosis of flourishing is made if someone feels one of the three hedonic well-being symptoms (items 1–3) “every day” or “almost every day” and feels six of the 11 positive functioning symptoms (items 4–14) “every day” or “almost every day” in the past month. A person is diagnosed as languishing if they feel the three hedonic well-being symptoms “never” or “once or twice” and six of the 11 eudaimonic well-being symptoms “never” or “once or twice” in the past month. Individuals not meeting neither “languishing” nor “flourishing” criteria are diagnosed as “moderately mentally healthy.”

Confirmatory Factor Analyses (CFA) were conducted to examine the structural validity of the Kazakhstani version of the MHC-SF with the *lavaan* (Rosseel, 2012) and *semPlot* (Epskamp, 2015) packages in R (R Core Team, 2020). Based on the theory and previous research (see Introduction section), five distinctive models were tested in this study: (1) a single factor model in which all the 14 items load into one general factor of well-being; (2) a two-factor model with two correlated factors: hedonic well-being (EWB, items 1–3), and eudaimonic well-being (SWB and PWB, items 4–14); (3) a three-factor model, with three correlated factors of well-being (EWB, items 1–3; SWB, items 4–8; PWB, items 9–14 PWB); (4) a second-order model were the three first order dimensions (EWB, SWB, PWB) load in a general factor of well-being; and (5) a bifactor model, with three separated factors of well-being (EWB, SWB, PWB) plus a general factor of well-being.

Participants with one or more missing data were excluded from the analysis. The Satorra-Bentler scaled chi-square test (SB χ^2^) test was used to evaluate the absolute fit of the model. However, because the SB χ^2^ test is considered highly conservative, additional absolute and incremental alternative fit indices were used to evaluate the model. Additional absolute fit indices included the SB χ^2^ to degrees of freedom ratio (SB χ^2^/*df*), the Root Mean Square Error of Approximation (RMSEA), the Standardized Root Mean Square Residual (SRMR), and the Akaike's Information Criterion (AIC). Indices of incremental fit comprised the Comparative Fit Index (CFI) and the Tucker-Lewis Index (TLI). Values of SB χ^2^/*df* <3, RMSEA and SRMR <0.06, and CFI and TLI > 0.95 indicated a good model fit, while SB χ^2^/*df* <5, RMSEA and SRMR <0.08, and CFI and TLI > 0.90 indicated a satisfactory fit (Hu and Bentler, 1999; Schreiber et al., 2006). In general, lower AIC values indicate a better fit.

The reliability of the factors of the best fitting model was estimated using omega (ω) coefficients using the *psych* package in R (Revelle, 2021). Omega has demonstrated to be superior to Cronbach's alpha and other reliability coefficients to capture the proportion of scale variance due to all common factors and the proportion of scale variance due to a general factor (Zinbarg et al., 2005, p. 132), as is the case for the multidimensional models tested in this study. Omega reliability coefficients (ω) were calculated to estimate the proportion of variance in the observed total score attributable to both general and specific well-being factors as suggested by Rodriguez et al. (2016). Furthermore, omega hierarchical (ω_h_) was calculated to estimate the proportion of variance in total scores attributable to a single general well-being factor (Rodriguez et al., 2016). Additionally, omega subscale (ω_s_) was used to calculate the reliability of the subscale scores and hierarchical subscale (ω_hs_) coefficients were used to estimate the amount of variance in each subscale that is explained by a specific factor (i.e., EWB, SWB, and PSW), after removing the reliable variance explained by the general well-being factor. Following Perreira et al. (2018), ω coefficients > 0.50 were considered satisfactory.

Gender and age invariance of the best-fitting factor model was examined using multigroup confirmatory factor analyses (MGCFA). We tested configural invariance, metric invariance, scalar invariance, and strict invariance across gender (male vs. female) and age (younger vs. older). Configural invariance was confirmed if RSMEA and SRMR were <0.08 and CFI was >0.95 (Cheung and Rensvold, 2002). A relative change of ≤ 0.010 in CFI, paired with a relative change of ≤ 0.015 in RMSEA and ≤ 0.030 in SRMR (for metric invariance) or ≤ 0.015 (for scalar and residual invariance) indicated support for measurement invariance (Cheung and Rensvold, 2002; Chen, 2007; Putnick and Bornstein, 2016).

## Results

### Descriptive Analysis

Descriptive statistics along with the percentage of participants meeting the criteria for the three categorical diagnoses of mental health in the Kazakhstani version of the MHC-SF are presented in Table 1. Skewness and kurtosis did not exceed 1 for any subscale. However, the Shapiro-Wilk test was statistically significant for all subscales, revealing that the data were assumed to be not normally distributed. Based on the diagnostic criteria of mental health as measured by the MHC-SF, the findings revealed that 19.4% of the sample were languishing, 49.8% were moderately mentally healthy, and 30.7% were flourishing. Relevant differences were found in the gender distribution for the categorical diagnosis of languishing, χ^2^ = 6.56, *p* = 0.038, with males (33.5%) demonstrating higher distributions for flourishing than females (24.7%). No statistically significant differences were observed with respect to age, χ^2^ = 1.68, *p* = 0.431, although a slightly lower proportion of younger students (under 20 years old) classified as languishing (16.2 vs. 19.6%) and a higher proportion as flourishing (29.2 vs. 25.9%) when compared to older students (20 years or older).

**Table 1:** Descriptive statistics.

	**M**	**SD**	**Skewness**	**Kurtosis**	**Shapiro-Wilks**
EWB	9.02	4.00	−0.40	−0.71	0.96^***^
SWB	10.78	6.29	0.20	−0.83	0.96^***^
PWB	17.45	7.80	−0.26	−0.92	0.97^***^
Total	37.03	16.26	−0.09	−0.82	0.98^***^
Mental health categories	Total	Female	Male	Younger	Older
Languishing	17.6%	18.1%	17.0%	16.2%	19.6%
Moderate	45.2%	48.0%	39.8%	45.4%	44.8%
Flourishing	27.9%	24.7%	33.5%	29.2%	25.9%

*EWB, emotional well-being; SWB, social well-being; PSW, psychological well-being*.

*** *p < 0.001*.

### Factor Structure of the MHC-SF

Confirmatory Factor Analyses (CFA) were conducted to examine the structural validity of the Kazakhstani version of the MHC-SF. The parameter estimates in the CFAs were obtained using the robust maximum likelihood (MLM) method to account for any deviations from normality (Brown, 2006). Table 2 presents the CFA fit indices for the five models tested in this study. As indicated by the SB χ^2^ values, none of the models fit perfectly. The single factor and the correlated two-factor models were found to have an absolute poor fit. The correlated three-factor and second-order factor models achieved satisfactory to good fit, with identical fitting indices as they are mathematically equivalent. The bifactor model demonstrated an overall good fit, with SB χ^2^/*df* = 3.16, CFI and TLI > 0.95, RMSEA and SRMR <0.06, and the lowest AIC.

**Table 2:** Confirmatory factor analysis fit statistics.

**Model**	**SB χ^**2**^_**(df)**_**	**SB χ^**2**^ / *df***	**CFI**	**TLI**	**RMSEA (90% CI)**	**SRMR**	**AIC**
Single factor	615.35^*^_(77)_	7.99	0.864	0.840	0.123 (0.114–0.132)	0.061	29,119.56
Two-factor	487.82^*^_(76)_	6.41	0.898	0.878	0.107 (0.098–0.117)	0.056	28,932.64
Three-factor	303.30^*^_(74)_	4.10	0.944	0.931	0.081 (0.071–0.090)	0.049	28,677.14
Second-order factor	303.30^*^_(74)_	4.10	0.944	0.931	0.081 (0.071–0.090)	0.049	28,677.14
Bifactor	199.64^*^_(63)_	3.16	0.967	0.953	0.067 (0.057–0.077)	0.030	28,554.91

*SB χ^2^, Satorra-Bentler scaled chi-square; df, degrees of freedom; CFI, comparative fit index; TLI, Tucker-Lewis Index; RMSEA, root square error of approximation; CI, confidence intervals; SRMR, standardized root mean square residual; AIC, Akaike's information criterion*.

* *wp < 0.001*.

**Figure 1:**
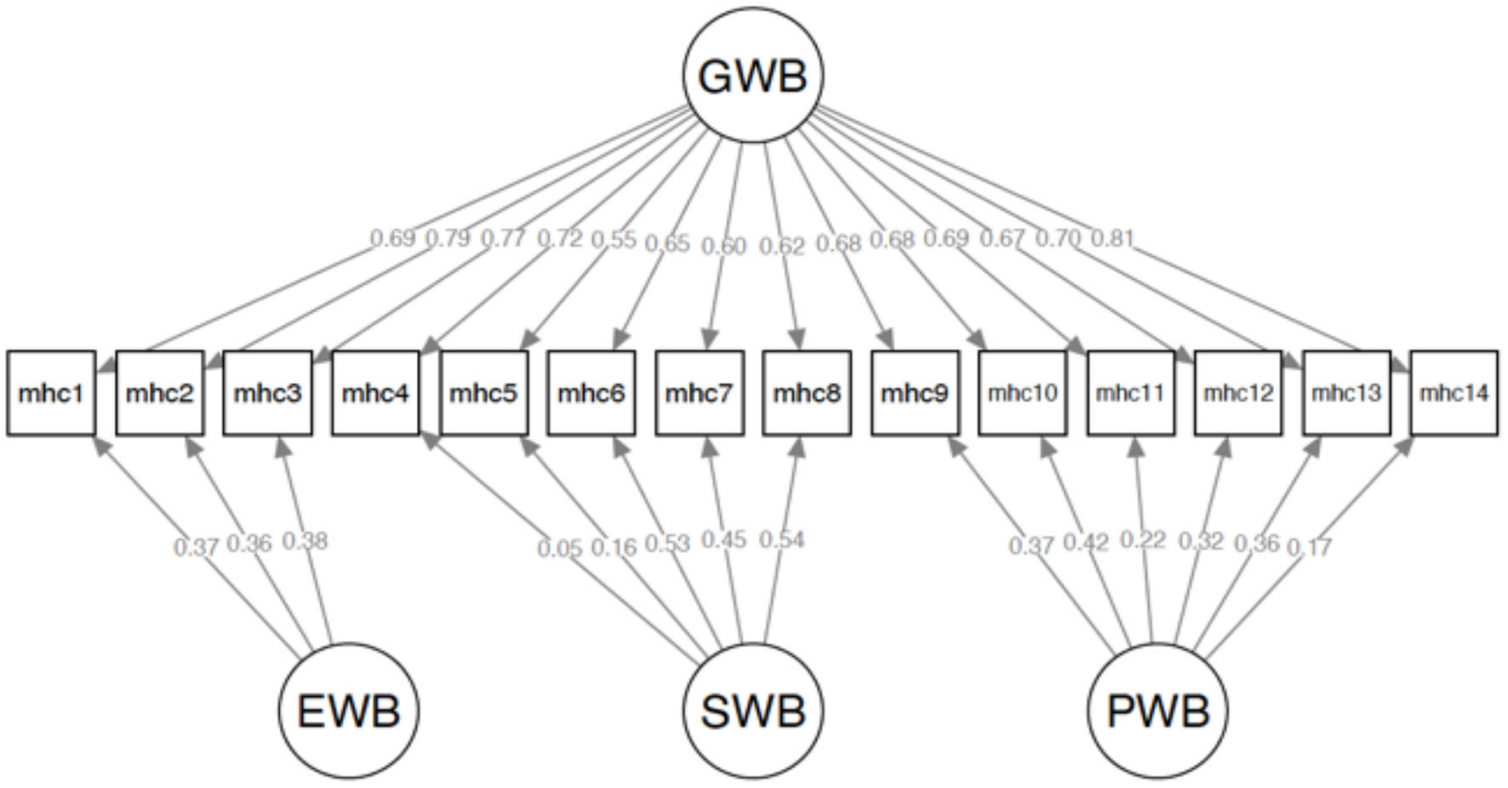
Standardized factor loadings of the bifactor model. GWB, general well-being; EWB, emotional well-being; SWB, social well-being; PSW, psychological well-being.

Substantial differences in SB χ^2^/*df*, TLI, CFI, RMSEA, and SRMR between the bifactor model and alternative models demonstrated the superiority of the bifactor model. Figure 1 presents the standardized path estimates for the bifactor model. All standardized path estimates loaded significantly in the general well-being factor (β = 0.55–0.81, *p* <. 001). Also, all items loaded significantly (*p* < 0.01) in the hypothesized specific factor, except for the item 4 (β = 0.05, *p* = 0.272). Noteworthy, all items exhibited higher loadings on the general factor than their respective specific factor (EWB, SWB, and PWB), indicating that the variances of the items were generally explained by the general well-being factor.

### Internal Consistency

The omega reliability coefficient indicated that 93% of the variance was explained by both the general and three specific wellbeing factors (ω = 0.93). The omega hierarchical coefficient (ω_h_) was 0.86, suggesting that 86% of the variance of uni-weighted total scores could be explained by individual differences on the general factor. Moreover, 92% of the reliable variance of the total scores could be attributed to the general well-being factor (ω_h_/ω = 0.92) and only 7% of the reliable variance can be attributed to the multidimensionality associated with the specific well-being factors (ω – ω_h_ = 0.07). Thus, raw total scores in the Kazakhstani version of the MHC-SF can be interpreted as unidimensional reflections of well-being.

Omega subscale coefficients indicated that 47% of the variance in the EWB subscale could be explained by EWB and the general factor (ω_ewb_ = 0.47), 64% of the variance in the SWB subscale could be explained by SWB and the general factor (ω_ewb_ = 0.64), and 75% of the variance in the PWB subscale could be explained by PWB and the general factor (ω_ewb_ = 0.75). However, the omega hierarchical subscale (ω_hs_) coefficients were low for the three well-being subscales (ω_h−ewb_ = 0.09, ω_h−swb_ = 0.15, ω_h−pwb_ = 0.12), indicating that the ability of the subscales of the Kazakhstani version of the MHC-SF to reliably measure specific variance of the three well-being components is low.

### Measurement Invariance

The MGCFA results for measurement invariance for the Kazakhstani version of the MHC-SF across gender (male vs. female) and age groups (under 20 year-old vs. 20+ year-old) are presented in Table 3. The bifactor model fitted the data satisfactorily across gender and age, indicating that configural invariance was supported for both variables (CFI > 0.95, RSMEA <0.08). Equality constraints were imposed on all factor loadings for both gender and domain groups to test full metric invariance. The ΔCFI and ΔRSMEA indicated full metric invariance (ΔCF < 0.01, ΔRSMEA <0.30). Equality constraints were then imposed on all intercepts and the three difference tests also indicated full scalar invariance. Finally, equality constraints were imposed on all residual variances, with the ΔCFI and ΔRSMEA supporting full strict invariance. These findings demonstrate the measurement invariance of the Kazakhstani version of the MHC-SF across gender and age.

**Table 3:** Measurement invariance of the bifactor model across gender and age.

**Model**	**SB χ^**2**^_**(df)**_**	**CFI**	**RMSEA**	**ΔCFI**	**ΔRMSEA**
**Gender invariance**					
Configural	319.11 _(126)_	0.965	0.069	–	–
Metric	347.32 _(150)_	0.964	0.064	0.001	0.005
Scalar	377.08 _(160)_	0.961	0.065	0.004	0.001
Strict	390.26 _(164)_	0.959	0.066	0.002	0.001
**Age invariance**					
Configural	357.71 _(126)_	0.958	0.076	–	–
Metric	400.64 _(150)_	0.955	0.072	0.003	0.004
Scalar	419.15 _(160)_	0.953	0.071	0.002	0.001
Strict	431.46 _(164)_	0.952	0.071	0.001	0.000

*SB χ^2^, Satorra-Bentler scaled chi-square; df, degrees of freedom; CFI, comparative fit index; RMSEA, root mean square error of approximation*.

^*^p < 0.001. Gender (n_male_ = 236, n_female_ = 425), Age (n_under20years−old_ = 383, n_20+years−old_ = 281).

## Discussion and Conclusions

The main goal of the present study was to examine the psychometric properties of the Kazakhstani version of the MHC-SF. We used descriptive analysis to examine the distribution of the scores and the prevalence of the categorical diagnoses of states of mental health. Confirmatory factor analysis techniques were implemented to evaluate and compare the fit of the well-being model proposed by the authors of the scale, which comprises three correlated factors representing emotional, social, and psychological well-being (Keyes et al., 2008), with alternative factorial solutions. We also explored the reliability of the scores using hierarchical omega statistics. Moreover, we tested whether the measure varied across different gender and age groups using multigroup confirmatory factor analysis.

Analysis of the distributions of categorical diagnosis of mental health states in our sample demonstrated that 17.6% of the participants reported being languishing, 45.2% moderately mentally healthy, and 27.9% flourishing. These estimates are similar to those found in previous studies with adult samples (Keyes et al., 2008; Lamers et al., 2011; Petrillo et al., 2015) but comparatively lower than those typically reported by younger populations in other contexts (e.g., Luijten et al., 2019). This could be because participants in our study are, on average, older than those in previous studies, and the probabilities of experiencing common psychological challenges increase through adolescence, reaching a peak in early adulthood (Kessler et al., 2007). Also, the data in the present study were collected in the early stage of the COVID-19 pandemic, which obviously contributed to the increased levels of psychological distress in our sample.

The original three-factor model displayed acceptable goodness-of-fit indexes, and comparatively better than those of single-factor and two-factor models. This is consistent with the results reported in previous validation studies of other versions of the MHC-SF (Lamers et al., 2011; Joshanloo et al., 2013; Karaś et al., 2014; Petrillo et al., 2015; Echeverría et al., 2017). However, the results revealed that a bifactor model provides a better representation of the factorial structure of the Kazakhstani version of the MHC-SF compared to the original model and other competing models. These results are consistent with a growing number of studies that suggest that the MHC-SF measures a predominant general well-being factor and three specific factors that correspond to the emotional, social, and psychological well-being subscales (De Bruin and Du Plessis, 2015; Jovanović, 2015; Hides et al., 2016; Echeverría et al., 2017; Rogoza et al., 2018; Longo et al., 2020).

An interesting finding in the present study was that item 4 (i.e., social contribution) had no salient loading on social well-being in the Kazakhstani version of the MHC-SF. Low factor loadings or no statistically significant loadings of item 4 on social well-being have been reported in previous studies using CFA and ESEM approaches in samples from Argentina (Perugini et al., 2017), New Zealand (Joshanloo et al., 2017), Iran (Joshanloo, 2016a), and Serbia (Joshanloo and Jovanović, 2017). Some authors have suggested that this may be because social contribution is normally seen as connected to more individual, private aspects of well-being as it refers to personal “feelings of usefulness” (Bobowik et al., 2015, p. 10). Relatedly, it has been proposed that social contribution may be a more accurate indicator of psychological well-being. In this sense, Joshanloo (2016b) argued that the belief that one can contribute to society is related to the perception that one has a series of facilitating psychological skills, such as positive relationships with others.

The strong loadings of all 14 items on the general well-being factor and the high omega reliability omega hierarchical coefficient (ω_h_) confirm the existence of a unitary/cohesive construct of general well-being that is reliably measured by the MHC-SF in the Kazakhstani context. This provides additional support to the proposition that a single overarching well-being construct could accurately integrate several conceptions of well-being (e.g., hedonia and eudaimonia) (Chen et al., 2013; Díaz et al., 2015; Disabato et al., 2016; Strelhow et al., 2020). However, there is relative agreement that the hedonic and eudaimonic components of well-being are related but distinct constructs and correlate differently with other predictors and outcomes (Gallagher et al., 2009; Delle Fave et al., 2011; Huta and Waterman, 2014). Therefore, future research should further explore the adequacy of using a total score to measure the general well-being using other measures and in other contexts.

Despite the good fit of the bifactor model, this study provides limited support for the multidimensionality of the Kazakhstani version of the MHC-SF. First, all items demonstrated statistically significant loadings to their expected specific factors, but these loadings were not substantial and were always lower relative to the general well-being factor. Second, the reliability of the scores was adequate to good for the social and psychological subscales, but low for the emotional subscale, as estimated by omega subscale coefficients (ω_s_). Third, the scores for the three well-being subscales explained little variance beyond that explained by the general factor, as indicated by the low omega hierarchical subscale coefficients (ω_hs_). This indicates that the ability of the Kazakhstani version of the MHC-SF subscales to reliably measure the specific variances of EWB, SWB and PWB is too low, as has been reported for other samples (e.g., Jovanović, 2015).

The findings of the study suggest that the bifactor model could be used to compare parameter estimates across genders. Moreover, an excellent fit was also found when the sample was split into a group of younger and older adults. These conclusions are aligned with previous studies in other contexts and further support the invariance of the structure of the MHC-SF bifactor model across different gender and age groups (e.g., Echeverría et al., 2017; Lamborn et al., 2018).

In sum, the results imply that a bifactor structure of the MHC-SF with a general well-being factor and three specific factors representing EWB, SWB, and PWB fits the data better than the original three factor structure and other competing models in the context of Kazakhstan. Data further supports the adequacy of the bifactor model of the MHC-SF in the sample, regardless of gender or age. The total score of the MHC-SF can be used as a reliable and valid indicator of general well-being that supports the diagnosis of participants as flourishing, moderately mentally healthy, or languishing. However, caution should be applied when using and interpreting the EWB, SWB and PSW subscale scores, as most of the reliable variance in subscale scores is attributable to the general well-being factor. This prevents the interpretation of the specific subscale scores of the Kazakhstani version of the MHC-SF as unequivocal indicators of EWB, SWB, and PSW. A plausible explanation is that the number of items in the MHC-SF could be insufficient to fully capture the complexity and breadth of well-being as a multidimensional construct with two or three correlated subscales—as proposed by the authors of the scale—without accounting for the presence of a general factor of well-being that represents the shared variance among all items (Jovanović, 2015; Longo et al., 2020). Moreover, the number of items in the MHC-SF is perhaps excessive for a single latent factor of well-being to fit adequately the data (Longo et al., 2020). However, it should be noted that bifactor models tend to overfit and set better than any other confirmatory models, regardless of the population's true model (Markon, 2019). Furthermore, bifactor models tend to produce less reliable specific factors that are well represented by their constituent indicators (Watts et al., 2019), as was the case in our study. Therefore, future studies should confirm the suitability of the bifactor structure beyond the interpretation of common fit statistics using innovative approaches, as proposed by Bonifay and Cai (2017).

In this study, we report several approaches to determine the psychometric characteristics of the Kazakhstani version of the MHC-SF. We conclude that this version of the MHC-SF is useful for examining a general factor of well-being and the measurement invariance of the instrument across University gender and age groups. However, we discourage the use of subscale scores as unequivocal indicators of emotional, social, and psychological well-being in this context. Some of the limitations of the present study are the use of self-reported data and the use of convenience University samples. Future research is needed on the dimensionality of Kazakhstani version of the MHC-SF in more representative samples. Another limitation is that the study does not examine the relationships between Kazakhstani version of the MHC-SF scores and convergent measures. Other studies should test the construct validity of the MHC-SF in Kazakhstan and similar contexts.

## Data Availability Statement

The raw data supporting the conclusions of this article will be made available by the authors, without undue reservation.

## Ethics Statement

The studies involving human participants were reviewed and approved by Nazarbayev University Institutional Research Ethics Committee (IREC). The patients/participants provided their written informed consent to participate in this study.

## Author Contributions

DH-T and LI contributed to the conception and design of the study, organized the database, performed the statistical analysis, and wrote the first draft of the manuscript. AM, NL, YN, AA, AC, and JS wrote the sections of the manuscript. All authors contributed to the article and approved the submitted version.

## Funding

This research was funded by the Nazarbayev University Faculty-Development Competitive Research Grants Program (Reference Number 240919FD3902).

## Conflict of Interest

The authors declare that the research was conducted in the absence of any commercial or financial relationships that could be construed as a potential conflict of interest.

## Publisher's Note

All claims expressed in this article are solely those of the authors and do not necessarily represent those of their affiliated organizations, or those of the publisher, the editors and the reviewers. Any product that may be evaluated in this article, or claim that may be made by its manufacturer, is not guaranteed or endorsed by the publisher.
